# Construction and optimization of a nisin-controlled expression vector using a pre-screened strong promoter in *Streptococcus thermophilus*

**DOI:** 10.3389/fmicb.2025.1586348

**Published:** 2025-08-18

**Authors:** Yanxin Ye, Ruiting Zhao, Leilei Li, Zhi Li, Yanyan Chen, Zhenshang Xu

**Affiliations:** ^1^School of Life Science and Engineering, Henan University of Urban Construction, Pingdingshan, China; ^2^School of Bioengineering, Qilu University of Technology, Shandong Academy of Science, Jinan, China

**Keywords:** *Streptococcus thermophilus*, nisin-controlled expression system, green fluorescent protein, strong promoter, improvement

## Abstract

The nisin-controlled gene expression (NICE) system is an efficient and promising gene expression system for *Lactococcus lactis*. To enhance the expression efficiency of the NICE system in *Streptococcus thermophilus* ATCC19258, an inducible expression vector, pNZ8148-PnisA-gfp-PnisR-nisR-nisK, containing the regulatory element NisR/K and the promoter PnisR, was first constructed using the basic plasmid pNZ8148. Green fluorescent protein (GFP), as the reporter protein, was cloned downstream of PnisA in the vector pNZ8148 to detect protein expression. The resulting expression vector was electroporated into *S. thermophilus*, *Lactobacillus plantarum*, and *Enterococcus faecium*, demonstrating that the NICE system can be used to induce protein production in various hosts of lactic acid bacteria. The optimal conditions for protein expression of the recombinant strain *S. thermophilus*/pNZ8148-PnisA-gfp-PnisR-nisR-nisK also showed that the expression level was the highest when the optimal induction concentration of nisin was 2,500 ng/mL for 3 h after induction. The recombinant plasmid pNZ8148-PnisA-gfp-PnisR-nisR-nisK was optimized using a strong promoter (P15, P18, P23, or P25) pre-screened from *S. thermophilus* instead of the native promoter PnisR. The results indicated that when the derived plasmid pNZ8148-PnisA-gfp-P25-nisR-nisK was electroporated into *S. thermophilus*, the resulting recombinant strain *S. thermophilus/*pNZ8148-PnisA-gfp-P25-nisR-nisK exhibited the highest expression level of heterologous green fluorescent protein. These results suggest that the improved plasmid-based nisin-controlled expression system has the potential to be used for desired protein production in *S. thermophilus*.

## 1 Introduction

*Streptococcus thermophilus*, a traditional starter, is extensively employed as an indispensable lactic acid bacterium (LAB) in industrial cheese and yogurt fermentation. It can serve as an attractive microbial cell factory for producing food ingredients and high-value metabolites, such as lactic acid, γ-aminobutyric acid, vitamins, and exopolysaccharides, because of its long history of safe usage and the importance of *S. thermophilus* in the food industry (Mu et al., 2020). In addition, the discovery of the inherent capabilities of *S. thermophilus* has greatly increased its potential as a new host for heterologous protein production (Lecomte et al., 2016; Zhao et al., 2023), providing the possibility of genetically engineered bacteria with plasmids or linear DNA fragments, similar to other *Lactococcus lactis strains* (Markakiou et al., 2023). However, the low production of intracellular or extracellular expression of biotechnological and biomedical proteins limits its practical application. Therefore, the availability of easy-to-operate and tightly controlled gene expression systems is critical for the development of these applications.

Currently, gene expression systems in *S. thermophilus* are still relatively scarce compared to other LAB (Markakiou et al., 2023), although several inducible gene expression systems, such as the tetracycline-inducible system, pheromone-induced expression system, food-grade vector-controlled expression system based on the α-complementation of β-galactosidase, nisin-controlled gene expression (NICE) system, and new Zn^2+^-controlled expression (ZICE) system (Blomqvist et al., 2006; Lecomte et al., 2016; Xu et al., 2022; Markakiou et al., 2023; Xu et al., 2024), have been developed for conditional gene expression in *S. thermophilus*. Among them, the NICE system, which consists of the inducible promoter PnisA and the dual signal transduction components NisR/K (NisK, the sensor kinase; NisR, the regulator protein) (Ni et al., 2017), is an efficient and promising food-grade expression system for LAB based on the auto-induction mechanism of nisin biosynthesis in *L. lactis* (de Ruyter et al., 1997). When the target gene is placed downstream of the PnisA promoter, effective gene expression can be induced by adding nisin to the culture medium (Soumya et al., 2023). Because of their adaptation to other LAB, NICE components have been integrated into various expression vectors. For example, a recombinant vector, pRSNPed using the NICE system, has been electrotransformed into *S. thermophilus* ST128, *L. lactis* subsp., and *Lacticaseibacillus casei*. Zones of inhibition were not observed when the transformant *S. thermophilus* was tested against *L. monocytogenes* NR30 by nisin induction compared to *L. lactis* and *L. casei* transformants (Renye and Somkuti, 2009). This phenomenon may be the result of inefficient expression of NisR/K regulatory elements (both NisR and NisK expression are driven by the promoter of PnisR) that are controlled in the plasmid by the original *L. lactis* promoter. This promoter may not function optimally in *S. thermophilus*, thus limiting the availability of NisR and NisK promoters. The selection of an appropriate native promoter from *S. thermophilus* is still an important consideration for optimizing the expression elements of the NICE system in *S. thermophilus* compared to those developed for other widely used LAB (Huang et al., 2023; Chaudhary et al., 2024). Thus, a promoter toolbox for the application of *S. thermophilus* and other LAB in the fields of metabolic engineering and synthetic biology was constructed in our previous study (data not published), and four strong promoters (the DNA sequences of promoters P15, P18, P23, and P25 are shown in Supplementary Table S1) of *S. thermophilus* were screened using RNA-seq and characterized using green fluorescent protein as the reporter protein.

The plasmid pNZ8148 is a popular vector for nisin-inducible expression of heterologous proteins in LAB, and the maximum protein expression level achievable with pNZ8148 is relatively low (Kazi et al., 2022). In this study, we constructed and optimized a nisin-controlled gene expression system in *S. thermophilus* by improving the plasmid pNZ8148. The resulting expression vector was electrotransformed into *S. thermophilus* ATCC19258, *Lactiplantibacillus plantarum* WCFS1, and *Enterococcus faecium* ATCC19434, and the functional properties of the expression system were studied using superfolder green fluorescent protein as a reporter in various LAB hosts. Furthermore, to enhance the expression of the heterologous protein, we replaced the PnisR promoter with strong selective promoters (P15, P18, P23, and P25) to improve the expression efficiency of the NisR/K regulatory elements and further optimize the nisin-induced expression system in *S. thermophilus*.

## 2 Materials and methods

### 2.1 Strains and plasmids

All plasmids and strains used in this study are listed in Table 1. *S. thermophilus* ATCC19258, *L. plantarum* WCFS1, and *E. faecium* ATCC19434 were stored in our laboratory and served as hosts for the protein expression. The host *Escherichia coli* XL1-Blue *strain* was used for the propagation of recombinant plasmids, and the vector pNZ8148 was used for plasmid improvement by NICE system integration.

**Table 1:** Strains and plasmids used in this study.

Strains or plasmids	Characteristics	Origin
*S. thermophilus* ATCC 19258	Host for expression	Our laboratory
*L. lactis* MG1363	Genomic DNA extraction as a template for amplification of key components promoter PnisR_,_ regulatory element NisR/NisK	Our laboratory
*L. plantarum* WCFS1	Host for expression	Our laboratory
*E. faecium* ATCC19434	Host for expression	Our laboratory
*E. coli* XL1-Blue	Host for cloning	Our laboratory
*S. thermophilus* NICE-PnisA	*S. thermophilus* containing plasmid pNZ8148-PnisA-gfp-PnisR-nisR-nisK	This study
*L. plantarum/*pNZ8148-PnisA-gfp-nisR-nisK	*L. plantarum* containing plasmid pNZ8148-PnisA-gfp-PnisR-nisR-nisK	This study
*E. faecium* /pNZ8148-PnisA-gfp-nisR-nisK	*E. faecalis* containing plasmid pNZ8148-PnisA-gfp-PnisR-nisR-nisK	This study
*S. thermophilus* NICE-P15	*S. thermophilus* containing plasmid pNZ8148-PnisA-gfp-P15-nisR-nisK	This study
*S. thermophilus* NICE-P18	*S. thermophilus* containing plasmid pNZ8148-PnisA-gfp-P18-nisR-nisK	This study
*S. thermophilus* NICE-P23	*S. thermophilus* containing plasmid pNZ8148-PnisA-gfp-P23-nisR-nisK	This study
*S. thermophilus* NICE-P25	*S. thermophilus* containing plasmid pNZ8148-PnisA-gfp-P25-nisR-nisK	This study
pNZ8148	Expression vector with the promoter PnisA	Used in this study
pNZ8148-PnisA-gfp	Vector pNZ8148 containing the GFP coding gene *GFP*	This study
pNZ8148-PnisA-gfp-PnisR-nisR-nisK	Recombinant plasmid pNZ8148-PnisA-gfp containing the promoter PnisR and two-component regulatory element NisR/NisK	This study
pNZ8148-PnisA-gfp-P15-nisR-nisK	Promoter P15 replaces PnisR in the plasmid pNZ8148-PnisA-gfp-PnisR-nisR-nisK	This study
pNZ8148-PnisA-gfp-P18-nisR-nisK	Promoter P18 replaces PnisR in the plasmid pNZ8148-PnisA-gfp-PnisR-nisR-nisK	This study
pNZ8148-PnisA-gfp-P23-nisR-nisK	Promoter P23 replaces PnisR in the plasmid pNZ8148-PnisA-gfp-PnisR-nisR-nisK	This study
pNZ8148-PnisA-gfp-P25-nisR-nisK	Promoter P25 replaces PnisR in the plasmid pNZ8148-PnisA-gfp-PnisR-nisR-nisK	This study

### 2.2 Chemical reagents

The 2 × Phanta Flash Master Mix, ClonExpress II one-step clone kit, Bacterial Genomic DNA Extraction Kit, and FastPure Gel DNA Extraction Mini Kit were provided by Nanjing Vazyme Biotech Co., Ltd., Nanjing, China. The TIANprep Mini Plasmid Kit was purchased from TIANGEN Biotechnology Co., Ltd., Beijing, China. The restriction enzymes QuickCut^™^ *NcoI* and *HindIII* were purchased from Takara Biomedical Technology Co., Ltd., Beijing, China. All other reagents used in this study were of analytical grade and were commercially available.

### 2.3 Experimental methods

#### 2.3.1 Construction of the recombinant nisin-induced expression vector

Primers used in this study are summarized in Supplementary Table S2. To express the *GFP* gene, the DNA fragment of the GFP-coding gene was PCR amplified from the plasmid pMN402 (Scholz et al., 2001) as a template using the primer pair GFP-NcoI-F/GFP-HindIII-R. The plasmid pNZ8148 (Kazi et al., 2022) was isolated from *E. coli* DH5α using the plasmid extraction kit (Tiangen Biochemical Technology Co., Ltd., Beijing, China) and linearized by the restriction endonucleases *Nco*I and *Hind*III. The purified PCR products of *GFP* and linearized vector pNZ8148 were ligated using the ClonExpress II one-step clone kit according to the manufacturer’s instructions, and the recombinant products were transformed into chemically competent *E. coli* XL1-Blue. Positive transformants were cultured and screened in LB solid medium containing 5 mg/mL chloramphenicol (0.25 g of chloramphenicol was dissolved in anhydrous ethanol, and the volume was fixed to 10 mL). The recombinant vector was isolated and sequenced from the positive transformants to generate the basic plasmid pNZ8148-PnisA-gfp.

DNA fragments of the PnisR-nisR-nisK containing complete nisR and nisK regulatory genes and the promoter of nisR/nisK were PCR amplified from the genomic DNA extracted from *L. lactis* MG1363 with primers (pNZ8148-gfp)-PnisR-F and (pNZ8148-gfp)-PnisR-R. The linear DNA of pNZ8148-PnisA-gfp was obtained by PCR amplification with the primers PN-gfp-F and PN-gfp-R using the recombinant plasmid pNZ8148-PnisA-gfp as a template. Purified PnisR-nisR-nisK and linearized vector pNZ8148-PnisA-gfp were ligated using the ClonExpress II one-step clone kit, and the recombinant products were transformed into chemically competent *E. coli* XL1-Blue. The recombinant plasmid pNZ8148-PnisA-gfp-PnisR-nisR-nisK with the NICE system was constructed and sequenced as described above.

#### 2.3.2 Improvement of the nisin-induced expression vector by replacing the promoter PnisR

The gene sequences of the four endogenous promoters (P15, P18, P23, and P25) of *S. thermophilus* and the primers used in this study are listed in Supplementary Tables S1, S2, respectively. These promoters were pre-screened by our laboratory from *S. thermophilus* cultured in LM17 and SM17 liquid media using RNA-seq technology, and functional characterization using GFP as a reporter protein confirmed the transcriptional activity of these four native promoters in *S. thermophilus* (data not published), according to previously reported methods (Kong et al., 2019; Wang et al., 2024). As crucial regulatory elements for gene expression, native promoters were used to optimize the nisin-inducible expression system.

To replace the promoter PnisR of the recombinant vector pNZ8148-PnisA-gfp-PnisR-nisR-nisK, a linear DNA fragment of pNZ8148-PnisA-gfp-nisR-nisK without the promoter PnisR was obtained by PCR amplification with primers PG1-F/PG1-R using the recombinant plasmid pNZ8148-PnisA-gfp-PnisR-nisR-nisK as the template. The inserted promoter DNA fragments P15, P18, P23, and P25 were PCR amplified from the genomic DNA of *S. thermophilus* using the primer pairs PG1-P15-F/PG1-P15-R, PG1-P18-F/PG1-P18-R, PG1-P23-F/PG1-P23-R, and PG1-P25-F/PG1-P25-R, respectively. The purified promoter-specific fragments of P15, P18, P23, and P25 were cloned into the linearized vector pNZ8148-PnisA-gfp-nisR-nisK and identified by PCR, generating recombinant plasmids pNZ8148-PnisA-gfp-P15-nisR-nisK, pNZ8148-PnisA-gfp-P18-nisR-nisK, pNZ8148-PnisA-gfp-P23-nisR-nisK, and pNZ8148-PnisA-gfp-P25-nisR-nisK, respectively. The identity of the recombinant plasmids was verified using nucleotide sequencing.

#### 2.3.3 Preparation of competent cells of *Streptococcus thermophilus* and transformation

The pre-cultured suspension of *S. thermophilus* ATCC19258 was transferred to fresh LM17 liquid medium at an inoculation volume of 2% (v/v) and cultured at 37°C for 12–16 h. When the OD_600nm_ of the culture reached 0.5, an equal volume of SGLM17 (LM17 broth with lactose 5.0 g/L, sorbitol 145.7 g/L, and glycine 200 g/L) was added and mixed evenly using a vortex oscillator, and then cultured for 1 h at 42°C without shaking. In addition, the pre-cultured suspensions of *L. plantarum* and *E. faecium* were transferred to fresh Sol I liquid medium (MRS broth with sorbitol 136.6 g/L and glycine 10 g/L) at an inoculation volume of 2% (v/v) and cultured at 37°C until the OD_600nm_ was 0.3–0.6. After culture, all the suspensions of *S. thermophilus* ATCC19258, *L. plantarum* WCFS1, and *E. faecium* ATCC19434 were placed in an ice bath for 30 min, centrifuged at 4°C for 6,000 r/min for 10 min to collect bacteria, and then resuspended in 1 mL SM Buffer (sucrose 326.0 g/L, MgCl_2_·6H_2_O 0.72 g/L), repeated three times. Competent cells of all strains were prepared by adding 50 μL of SM buffer. The resultant recombinant plasmids were electroporated into competent *S. thermophilus*, *L. plantarum*, or *E. faecium* to generate the corresponding recombinant strains, according to previously reported methods (Xu et al., 2022; Xu et al., 2024).

#### 2.3.4 Induced expression of heterologous protein by the NICE system in *Streptococcus thermophilus*

The recombinant strains (*S. thermophilus*, *L. plantarum*, and *E. faecium*) with a nisin-controlled expression vector were cultured at 42°C for 12–16 h in LM17 medium with 5 μg/mL chloramphenicol and then transferred into fresh LM17 medium at a rate of 2% (v/v) for heterologous protein expression. To determine the appropriate induction conditions of the combination for recombinant vector and host *S. thermophilus*, when the cell concentration of *S. thermophilus* reached 0.5–0.6 at OD_600nm_, 25 ng/mL nisin (nisin was dissolved in 0.02 M HCl and finally fixed to 10 mL, then filtered by a 0.2 μm filter membrane and stored at 4°C) was added and cultured for 0, 1, 2, 3, 4, 5, and 6 h to determine the optimal induction fermentation time. The effect of adding different concentrations of nisin at 0, 5, 10, 25, 50, 100, 250, 250, 500, 1,000, 2,500, 5,000, 10,000, 25,000, and 50,000 ng/mL on the expression of heterologous proteins in recombinant *S. thermophilus* was also studied to determine the optimal induced concentration of nisin without changing other conditions.

Meanwhile, the improved NICE system also induced the expression of the heterologous GFP protein. The fluorescence intensity was measured after culturing with 2,500 ng/mL nisin for 3 h when the cell concentration reached 0.5–0.6 at OD_600nm_, without nisin as the control. The other methods have been described previously.

#### 2.3.5 Determination of relative fluorescence units

The relative fluorescence units (RFU) were determined by the method described previously (Xu et al., 2024). After nisin induction, bacterial cells in different fermentation broths were harvested by centrifugation at 8000 rpm for 2 min, washed three times, and resuspended in phosphate-buffered saline (PBS). Then, 200 μL of the bacterial suspension was added to a 96-well plate, and the whole-cell fluorescence intensity was measured using a spectrofluorometer (Biotek, United States) by excitation at 485 nm and emission at 528 nm. The relative fluorescence units (RFU) were calculated as the fluorescence intensity/OD_600nm_.

### 2.4 Statistical analysis

All experiments were conducted in triplicate. SPSS Statistics 24.0 was used for data analysis in this study. Data are presented as the mean ± standard deviation (SD). Statistical differences between groups were analyzed using a two-tailed Student’s *t*-test. Asterisks indicate significant differences (^*^p < 0.05, ^**^p < 0.01, and ^***^p < 0.001), whereas ns indicates non-significant statistical differences.

## 3 Results

### 3.1 Construction of recombinant plasmid

**Figure 1:**
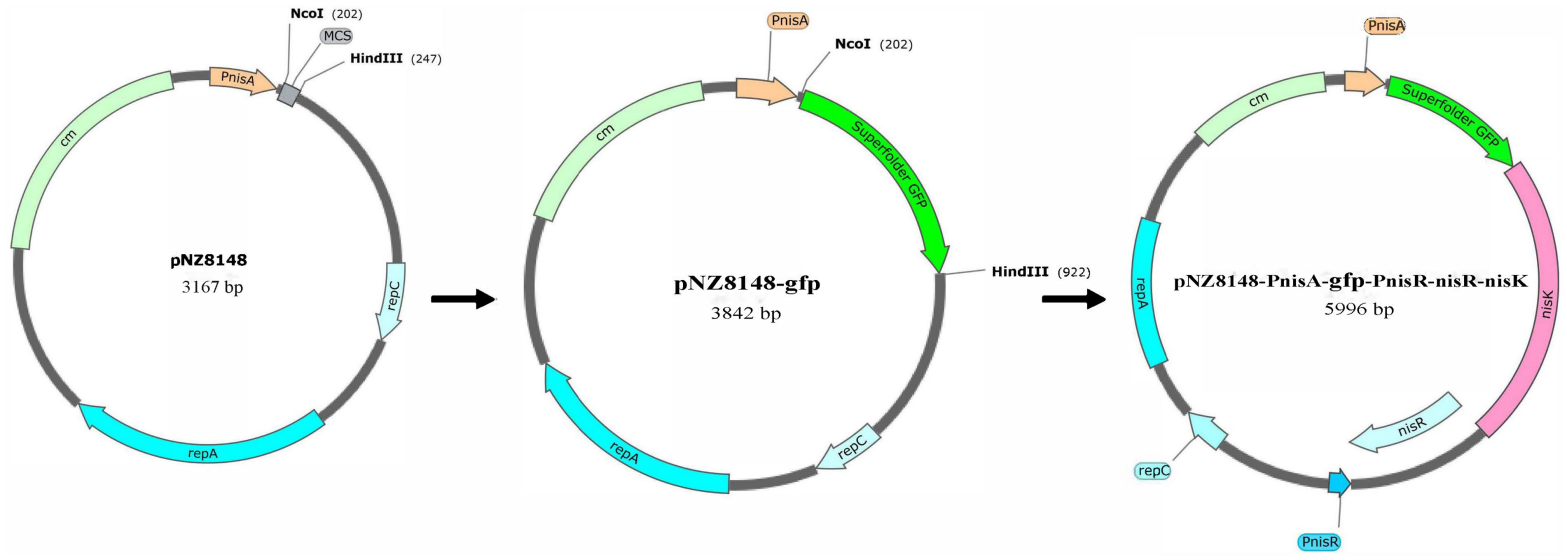
Schematic diagram of construction of the recombinant pNZ8148-PnisA-gfp-PnisR-nisR-nisK vector.

Green fluorescent protein (GFP) can be used as a reporter protein to quantify promoter activity because of its rapid and simple *in vivo* detection (Scholz et al., 2001). In this study, the nisin-controlled expression system of the recombinant pNZ8148-PnisA-gfp-PnisR-nisR-nisK vector was detected using super-folder GFP as a reporter protein, and PnisA was used as the nisin-induced promoter. As shown in Figure 1, the GFP-coding gene *GFP*, which was amplified with the primers pair GFP-NcoI-F/GFP-HindIII-R, using plasmid pMN402 as a template (Supplementary Figure S1, Lane 1–2), was first cloned downstream of the promoter PnisA of the plasmid pNZ8148 digested by the restriction endonucleases *NcoI-F* and *HindIII*. The linearized gene pNZ8148-PnisA-gfp was obtained by PCR amplification using the primers PN-gfp-F and PN-gfp-R (Supplementary Figure S1, Lane 5–6). The inserted gene fragment PnisR-nisR-nisK containing complete nisR/nisK regulatory genes and the PnisR/nisK promoter PnisR was amplified by primers (pNZ8148-gfp)-P_nisR_-F and (pNZ8148-gfp)-P_nisR_-R with *L. lactis* genomic DNA as the template (Supplementary Figure S1, Lane 3–4). The recombinant plasmid pNZ8148-PnisA-gfp-PnisR-nisR-nisK was successfully constructed using the ClonExpress II one-step clone kit according to a previous design (Figure 1).

### 3.2 Comparison of GFP expression by nisin induction in *Streptococcus thermophilus*, *Lactobacillus plantarum*, and *Enterococcus faecium*

To demonstrate whether the NICE system of pNZ8148-PnisA-gfp-PnisR-nisR-nisK can be used to regulate the production of target genes in LAB, the expression vector was introduced by electroporation into the host *S. thermophilus*, *L. plantarum*, and *E. faecium*, and the expression ability of the reporter gene was evaluated by detecting fluorescence intensity.

**Figure 2:**
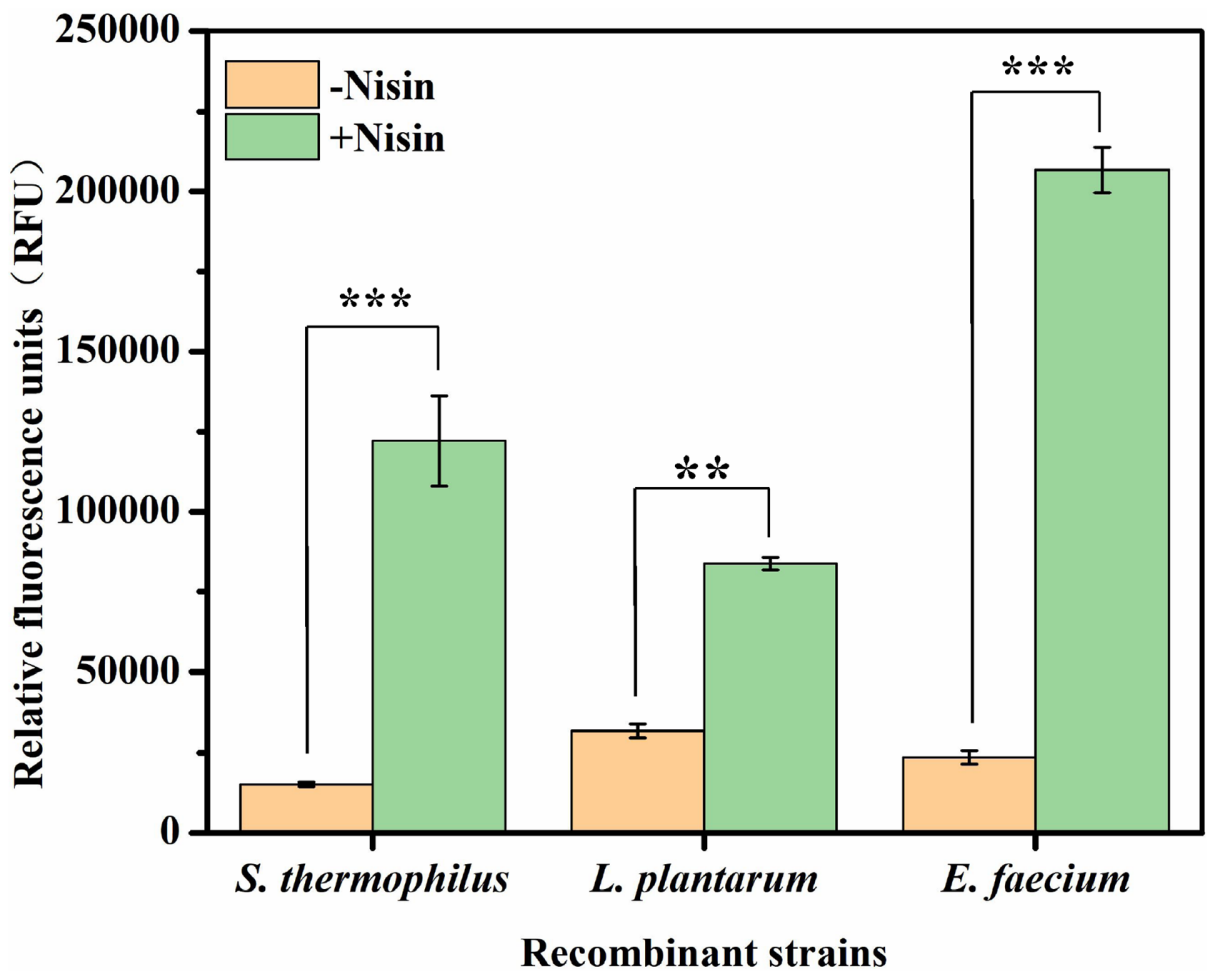
Expression levels of GFP in different recombinant *S. thermophilus* ATCC19258, *L. plantarum* WCFS1, and *E. faecium* ATCC19434 after induction with 25 ng/mL nisin for 3 h. −Nisin, control without nisin supplementation; +nisin, induced with 25 ng/mL nisin. Error bars show the standard deviations from three independent replicates. ^**^p < 0.01 and ^***^p < 0.001.

The recombinant strains containing the regulatory nisR/K +genes integrated in the vector were cultured in LM17 medium with 5 μg/mL chloramphenicol at 42°C for 12–16 h and then transferred to fresh LM17 medium at a ratio of 2% (v/v). Nisin induction is most preferred when the density of cultured cells reaches 0.4–0.5 at OD_600_, and the cells are in the exponential phase (Mierau et al., 2005). The fluorescence intensity was measured after incubation with 25 ng/mL nisin for 3 h when the cell concentration at OD_600_ reached 0.5–0.6. As shown in Figure 2 and Supplementary Table S3, the relative fluorescence intensity produced by different recombinant strains under nisin induction was obvious in all three kinds of lactic acid bacteria (−nisin as the experimental control). Specifically, the measured fluorescence intensities were 122170.23 ± 14142.87 RFU for recombinant *S. thermophilus*, 83797.62 ± 1942.08 RFU for recombinant *L. plantarum*, and 206759.68 ± 7071.07 RFU for recombinant *E. faecium*, respectively. These results demonstrate that the nisin-controlled expression system is not only functional in *L. plantarum* and *E. faecium* but also exhibits significant efficiency in initiating heterologous protein expression in *S. thermophilus*.

### 3.3 Time- and dose-dependent expression of GFP by nisin induction in *Streptococcus thermophilus*

**Figure 3:**
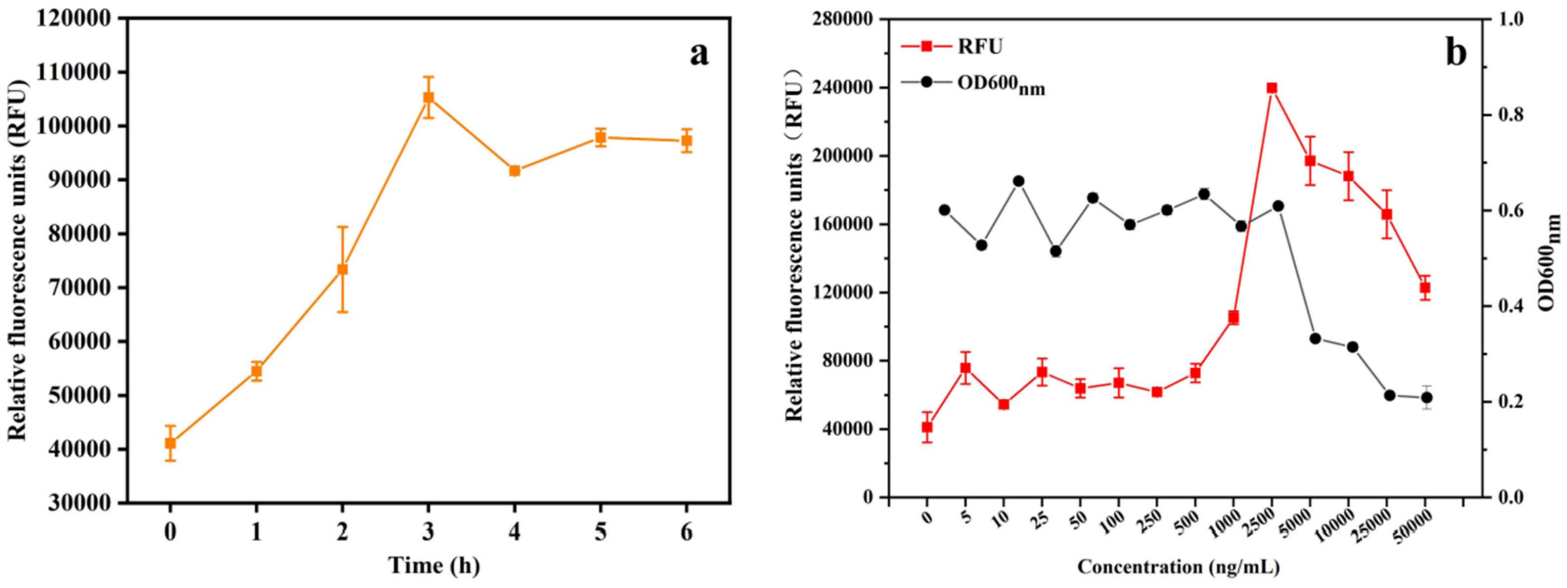
Optimum conditions for nisin-induced heterogeneous expression of GFP in recombinant *S. thermophilus*. **(a)** Effect of different induction times (0, 1, 2, 3, 4, 5, and 6 h) on GFP expression levels in *S. thermophilus* under 25 ng/mL nisin induction. **(b)** Relative fluorescence units (RFU) and OD_600nm_ values of cell density (measured as 600 nm) in the fermentation broth after induction with different doses of the inducer nisin for 3 h. The error bars show the standard deviations from three independent replicates.

To determine the time course of GFP expression, recombinant *S. thermophilus*/pNZ8148-PnisA-gfp-P_nisR_-nisR-nisK was cultured in LM17 medium with 25 ng/mL nisin for 0, 1, 2, 3, 4, 5, and 6 h. The results are shown in Figure 3a. The expression level of GFP protein increased over time, and the highest expression level of GFP protein was detected approximately 3 h after induction. However, when the induction time was longer than 3 h, the expression of protein decreased slightly.

Similar to other bacteria, protein expression levels depend on the amount of nisin used for induction. The correlation between nisin concentration and induced expression level was also tested to determine the optimal induction dosage of the inducer nisin. When the OD_600nm_ of the bacterial suspension was 0.5, induction was performed by adding different concentrations of nisin, and the expression of GFP was measured. As shown in Figure 3b, when 5 ng/mL to 1,000 ng/mL nisin was added at the time of inoculation, the RFU (relative fluorescence units) was significantly increased after induction for 3 h. The highest expression level was observed at a nisin concentration of 2,500 ng/mL with a fluorescence intensity of 239,770 ± 1,343 RFU. However, the result of cell growth monitored at OD_600nm_ showed that the growth of *S. thermophilus* was also affected by the increase in nisin concentration and was significantly inhibited when the addition of nisin was higher than 2,500 ng/mL. Therefore, the addition of 2,500 ng/mL nisin was shown to be the optimal concentration for the induction of protein expression in *S. thermophilus*, and strain growth was less affected. This effectively induced the expression of heterologous proteins using the nisin-controlled expression system, although the growth rate of the strain decreased at this concentration.

### 3.4 Optimization of the NisR/K-induced expression vector by replacing the promoter PnisR

**Figure 4:**
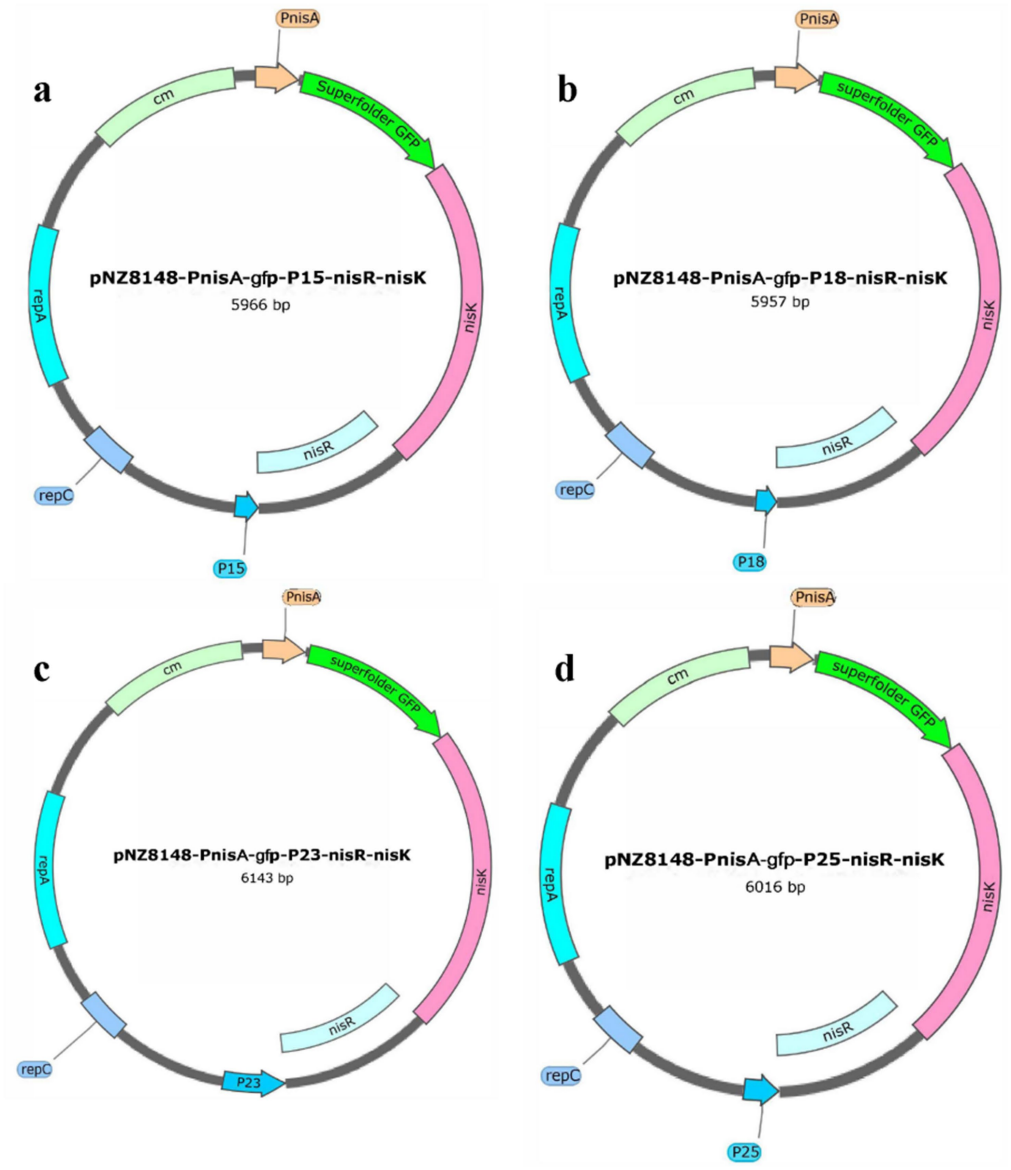
Schematic diagram of the recombinant plasmids pNZ8148-PnisA-gfp-P15-nisR-nisK **(a)**, pNZ8148-PnisA-gfp-P18-nisR-nisK **(b)**, pNZ8148-PnisA-gfp-P23-nisR-nisK **(c)**, and pNZ8148-PnisA-gfp-P25-nisR-nisK **(d)** by replacing different promoters.

To enhance the heterologous protein production of *S. thermophilus* using the NICE system, we attempted to replace the promoter PnisR in the NICE vector to verify whether the low protein production of *S. thermophilus* was the result of low expression of NisR/K regulatory elements. In our previous study, strong promoters (P15, P18, P23, and P25) from *S. thermophilus* were screened using RNA-seq and characterized using green fluorescent protein as the reporter protein (data not published). Thus, the four native promoters P15, P18, P23, and P25 from *S. thermophilus* were selected to replace PnisR in pNZ8148-PnisA-gfp-PnisR-nisR-nisK for recombinant plasmid optimization in this study. As shown in Figure 4, the recombinant plasmids pNZ8148-PnisA-gfp-P15-nisR-nisK (Figure 4a), pNZ8148-PnisA-gfp-P18-nisR-nisK (Figure 4b), pNZ8148-PnisA-gfp-P23-nisR-nisK (Figure 4c), and pNZ8148-PnisA-gfp-P25-nisR-nisK (Figure 4d) were successfully constructed according to the schematic structure using the same method as described above. The recombinant plasmids isolated from positive transformants were identified with designed specific primer pairs. PG1-P15-F/PG1-P15-R, PG1-P18-F/PG1-P18-R, PG1-P23-F/PG1-P23-R, and PG1-P25-F/PG1-P25-R, respectively, and the fragment sizes were consistent with the theoretical values, as shown in Supplementary Figure S2. These plasmids were then genetically sequenced correctly, indicating that all promoter genes had successfully replaced the original promoter PnisR.

### 3.5 Nisin induction of GFP expression by the optimized promoter of nisR/K

The constructed expression vectors of pNZ8148-PnisA-gfp-P15-nisR-nisK, pNZ8148-PnisA-gfp-P18-nisR-nisK, pNZ8148-PnisA-gfp-P23-nisR-nisK, and pNZ8148-PnisA-gfp-P25-nisR-nisK with different strength promoters were introduced by electroporation into *S. thermophilus*, and the positive recombinant strains containing different recombinant plasmids pNZ8148-PnisA-gfp-P15-nisR-nisK, pNZ8148-PnisA-gfp-P18-nisR-nisK, pNZ8148-PnisA-gfp-P23-nisR-nisK, or pNZ8148-PnisA-gfp-P25-nisR-nisK (named as *S. thermophilus* NICE*-*P15, *S. thermophilus* NICE*-*P18, *S. thermophilus* NICE-P23, and *S. thermophilus* NICE-P25, respectively) were selected by their resistance to chloramphenicol and PCR identification.

**Figure 5:**
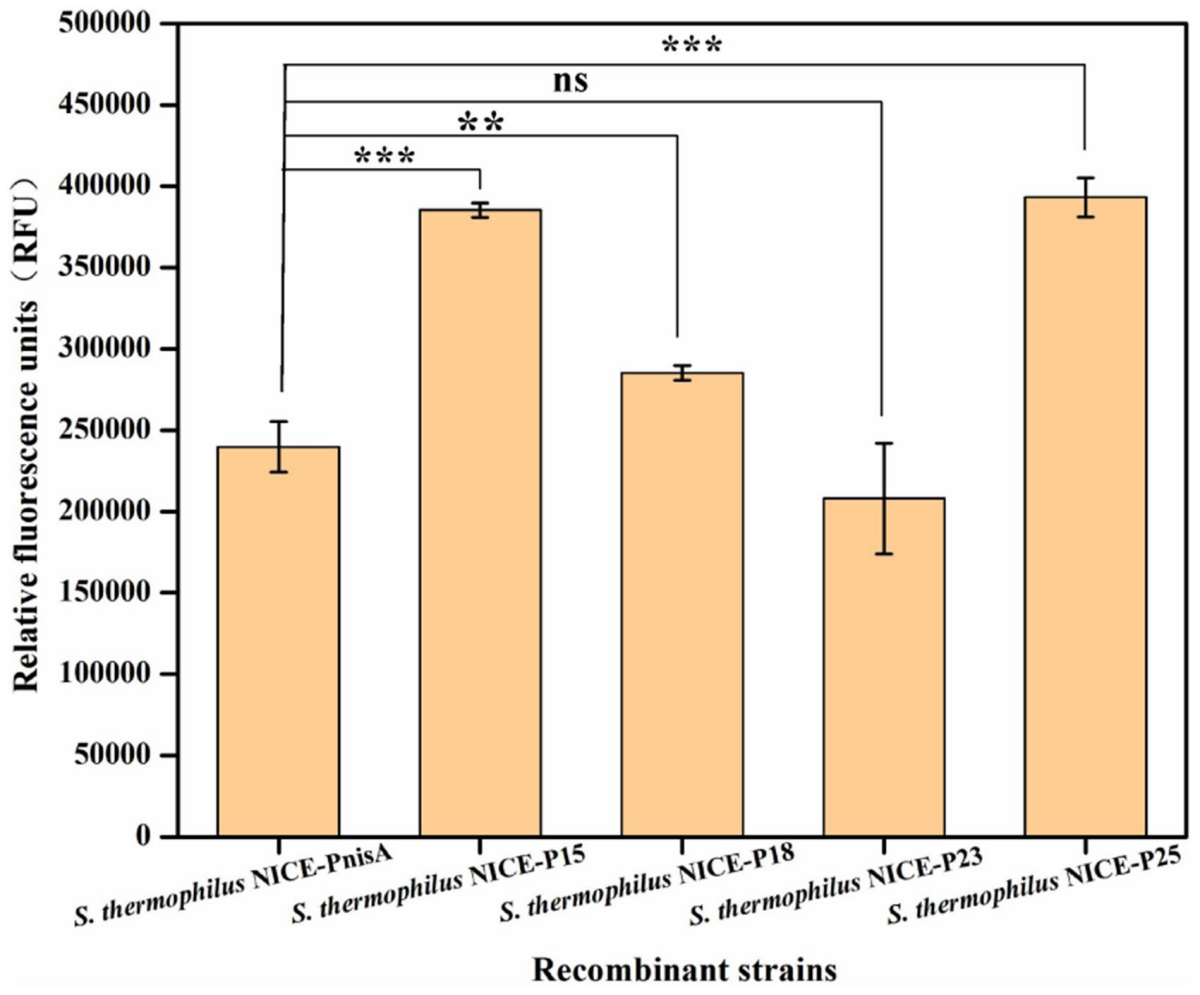
Expression levels of GFP in different recombinant strains containing the recombinant vector by replacing different promoters after induction with 2,500 ng/mL nisin for 3 h when the cell concentration reached 0.5 at OD_600nm_. The error bars show the standard deviations from three independent replicates. ^**^p < 0.01 and ^***^p < 0.001, and ns indicates non-significant statistical differences.

To investigate whether the expression level of the optimized nisin-induced expression vector in the recombinant strain *S. thermophilus* was improved, the recombinant strains were cultured at 42°C for 12–16 h in LM17 medium with 5 μg/mL chloramphenicol and then transferred into fresh LM17 medium at a rate of 2% (v/v). When the cell concentration reached 0.5–0.6 at OD_600nm_, the fluorescence intensity was measured after culture with 2,500 ng/mL nisin for 3 h. The results of the relative fluorescence intensity produced by different recombinant strains induced by nisin are shown in Figure 5 and Supplementary Table S4. All four recombinant strains produced fluorescence, among which *S. thermophilus*/pNZ8148-PnisA-gfp-PnisR-nisR-nisK (named *S. thermophilus* NICE*-*PnisA) was the control, and the fluorescence intensity was 239,761 ± 15,473 RFU. The fluorescence intensity produced by *S. thermophilus* NICE*-*P15, *S. thermophilus* NICE*-*P18, *S. thermophilus* NICE-P23, and *S. thermophilus* NICE-P25 was 385,323 ± 4,360 RFU, 285242 ± 4,551 RFU, and 207,942 ± 34028RFU, and 393,186 ± 12,139 RFU, respectively. Especially, the expression production of the recombinant strain *S. thermophilus* NICE-P25 was significantly enhanced compared to before the expression vector optimization, indicating that the NICE system of *S. thermophilus* was further optimized by replacing the nisR/K promoter of the expression vector, and the heterologous protein expression production was increased.

## 4 Discussion

*S. thermophilus*, the second most important starter after *L. lactis*, is widely used in the dairy industry and many traditional fermented products (Roux et al., 2022). Moreover, *S. thermophilus* is considered a good candidate for heterologous protein production (Lecomte et al., 2016; Huang et al., 2023). The NICE system has been extensively used in the food-grade bacterium *L. lactis* subsp. (Abedin et al., 2023), and the maximum level of recombinant protein expression under the control of this system was different in various LAB hosts (Douillard et al., 2011; Chaudhary et al., 2024). Several nisin-inducible systems have been developed for efficient gene expression in engineered hosts, but this host specificity has limited industrial applicability in *S. thermophilus* (Kazi et al., 2022; Barroso et al., 2023). The construction of plasmids and their application in microbial metabolic engineering are very common in biotechnological applications. A widely accepted and commercially developed plasmid, pNZ8148, serves as a food-grade vector (Soumya et al., 2023). Therefore, we aimed to develop an expression vector using the pNZ8148 plasmid for the production of heterologous proteins in *S. thermophilus* that allows the expression of recombinant genes in a regulated and improved manner compared to the level normally produced by the host. In this study, a recombinant vector pNZ8148-PnisA-gfp-PnisR-nisR-nisK induced by nisin was constructed, which contains both regulatory elements nisR and nisK, as well as the promoter nisR, and has a wider range of hosts. Thus, the expression vector was introduced by electroporation into *S. thermophilus* ATCC19258, *E. faecium* ATCC19434, and *L. plantarum* WCFS1. The results demonstrated that the single-vector NICE system functions in *S. thermophilus*, *E. faecium*, and *L. plantarum*, inducing the expression of green fluorescent protein, suggesting that the NICE system can regulate the production of target proteins in various LAB hosts. Nevertheless, we observed in our experiments (Figure 2) that the relative fluorescence intensity of *S. thermophilus* was lower than that of *E. faecium* and slightly higher than that of *L. plantarum*, which is similar to the results of Renye and Somkuti (2009). This may be caused by inefficient expression of the NisR/K regulatory elements in *S. thermophilus*, which are controlled by a natural *L. lactis* promoter in pNZ8148-PnisA-gfp-PnisR-NisR-nisK. The effects of induction time and nisin concentration on the NICE system in *S. thermophilus* for optimal gene expression levels were also investigated, and the results demonstrated that the highest GFP expression was achieved after induction with 2,500 ng/mL nisin for 3 h (Figure 3). Regrettably, even under identical fermentation conditions (induced with 25 ng/mL nisin for 3 h), there were differences in the GFP expression levels of recombinant *S. thermophilus* between different batches of experiments (Figures 3a,b), which may be caused by plasmid segregational instability and has been illustrated in *L. lactis* (Garay-Novillo et al., 2019; Kiewiet et al., 1993). Additionally, the pNZ series of vectors is a rolling circle replicating plasmid, and generally, their single-stranded intermediate contributes to their plasmid segregational instability during replication (Kazi et al., 2022). Consequently, the inherent instability of the recombinant plasmid during prolonged cultivation in the host represents a fundamental limitation of this study, which greatly restricts its potential for industrial application. For this reason, the stability of recombinant vector pNZ8148-PnisA-gfp-PnisR-NisR-nisK in *S. thermophilus* grown in LM17 medium will be tested in subsequent experiments and optimized, including adjusting the medium components and pH and optimizing the fermentation temperature and rotational speed, as well as exploring the replacement strategies of replicators to enhance the stability of the recombinant plasmid.

Previous studies have shown that various effective genetic tools, including plasmids, promoters, and selection markers, can now be used to achieve heterologous expression in *S. thermophilus* (Blomqvist et al., 2006; Kazi et al., 2022; Zhao et al., 2023). In particular, promoter selection is crucial for heterologous expression (Lecomte et al., 2016). To enhance the application potential of the nisin-inducible vector pNZ8148-PnisA-gfp-PnisR-nisR-nisK, we replaced the native PnisR promoter with four pre-screened endogenous promoters (P15, P18, P23, and P25), which were previously identified in *S. thermophilus* using RNA-seq technology and functionally characterized in *S. thermophilus* with GFP as a reporter protein according to previously reported methods (Wang et al., 2024). Experimental results showed that the relative fluorescence intensity of the recombinant strains *S. thermophilus* NICE*-*P15, *S. thermophilus* NICE*-*P18, and *S. thermophilus* NICE-P25 increased by 60.71, 18.96, and 63.99%, respectively, compared to the strain *S. thermophilus* NICE*-*PnisA. However, the protein expression of *S. thermophilus* NICE-P23 decreased by 13.27%. Therefore, promoter P25 is a good candidate for the dual signal transduction components NisR/K expression in the vector pNZ8148-PnisA-gfp-P25-nisR-nisK, which can effectively promote the expression of heterologous proteins.

Nisin induction in recombinant *S. thermophilus* NICE-P25 was performed as previously described (Soumya et al., 2023). It has been reported that a concentration of nisin in the range of 5–50 ng/mL can effectively induce protein expression in LAB (Renye and Somkuti, 2009; Wang et al., 2014; Tirziu et al., 2024), except for *L. lactis* NZ9000, for which the final concentration of nisin for protein-induced expression was 5 ng/L (Douillard et al., 2011). In any case, the concentration of nisin required to induce gene expression does not exceed the maximum level (250 ng/mL) allowed by the production standard (Renye and Somkuti, 2015). Nevertheless, when the concentration of nisin used in this study was 2,500 ng/mL, it was able to induce the highest expression of the recombinant gene in recombinant *S. thermophilus*, which may be caused by the promoter PnisA rather than the original promoter of *S. thermophilus*. Modification of the PnisA promoter in a plasmid containing the NICE system has been reported to increase gene expression induced by nisin in *L. lactis* IL1403 (Kim and Mills, 2007). Thus, we further attempted to modify the promoter PnisA or screen novel promoters to replace PnisA to improve the induced expression level of the plasmid in the host.

In summary, we successfully constructed a nisin-inducible expression vector (pNZ8148-PnisA-gfp-P25-nisR-nisK) using the pre-screened strong promoter P25 for *S. thermophilus*, which is also suitable for other lactic acid bacterial hosts. Our results also suggest that the plasmid-based nisin-controlled expression system can effectively promote the expression of heterologous proteins and has the potential for its application, providing a foundation for further optimization of the expression system in *S. thermophilus*.

## Data Availability

The original contributions presented in the study are included in the article/Supplementary material, further inquiries can be directed to the corresponding authors.
