# Supplementary material for: Construction and optimization of a nisin-controlled expression vector using a pre-screened strong promoter in Streptococcus thermophilus
Source: Front Microbiol. 2025 Aug 18;16:1586348. doi: 10.3389/fmicb.2025.1586348 (PMC12401007; doi:10.3389/fmicb.2025.1586348)
Supplement: Supplementary file 1 [file Supplementary_file_1.docx]

**Construction and Optimization of a Nisin-controlled Expression Vector using Pre-screened Strong Promoter in *Streptococcus thermophilus***

**Yanxin Ye^1^, Ruiting Zhao^2^, Leilei Li^1^, Zhi Li^1^, Yanyan Chen^1^*, Zhenshang Xu^2*^**

^1^ School of Life Science and Engineering, Henan University of Urban Construction, Pingdingshan, 467036, Henan, P.R. China;

^2^ School of Bioengineering, Qilu University of Technology, Shandong Academy of Science, Jinan, 250353, Shandong, P.R. China

**^∗^Corresponding author**: Yanyan Chen, E–mail: [20201006@huuc.edu.cn](mailto:yeyanxin2018@huuc.edu.cn)

Zhenshang Xu, E–mail: [xuzhenshang@126.com](mailto:xuzhenshang@126.com)

**Figure legends:**

# Supplementary Fig. S1 **:** PCR analysis of target genes.

**Supplementary Fig. S2:** Identification of the plasmid pNZ8148-PnisA-gfp-P15-nisR-nisK, pNZ8148-PnisA-gfp-P18-nisR-nisK, pNZ8148-PnisA-gfp-P23-nisR-nisK, and pNZ8148-PnisA-gfp-P25-nisR-nisK by PCR assay.

# Supplementary Fig. S1

###
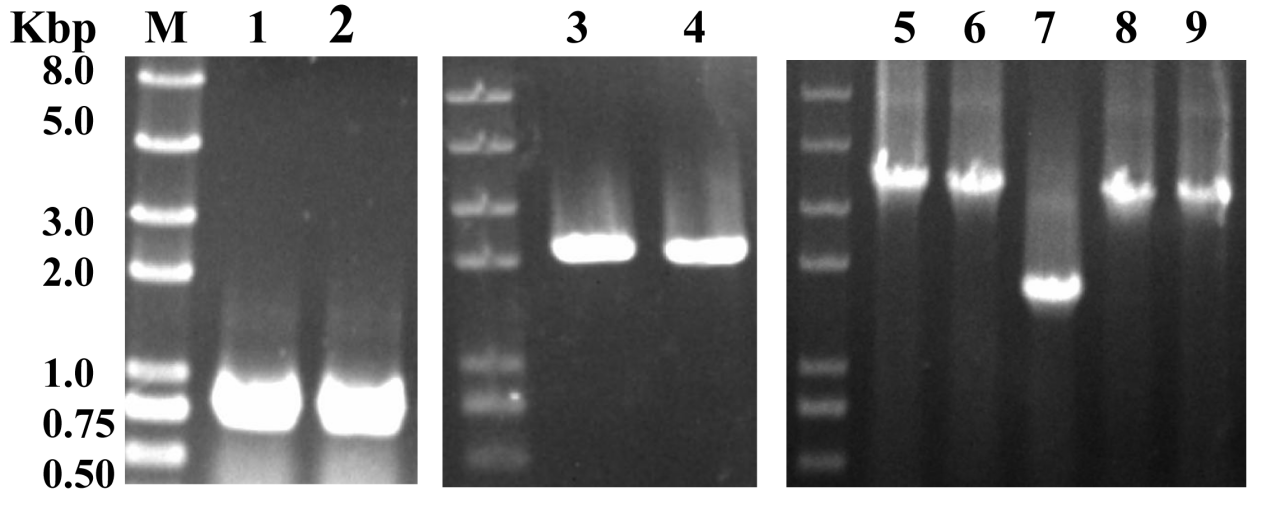


**Supplementary Fig. S1** PCR analysis of target genes. Lane M: DNA marker; Lane 1-2: PCR amplification products of *gfp* gene; Lanes 3-4: PCR amplification products of PnisR-nisR-nisK; Lanes 5-6: PCR amplification products of pNZ8148-PnisA-gfp.

# Supplementary Fig. S2


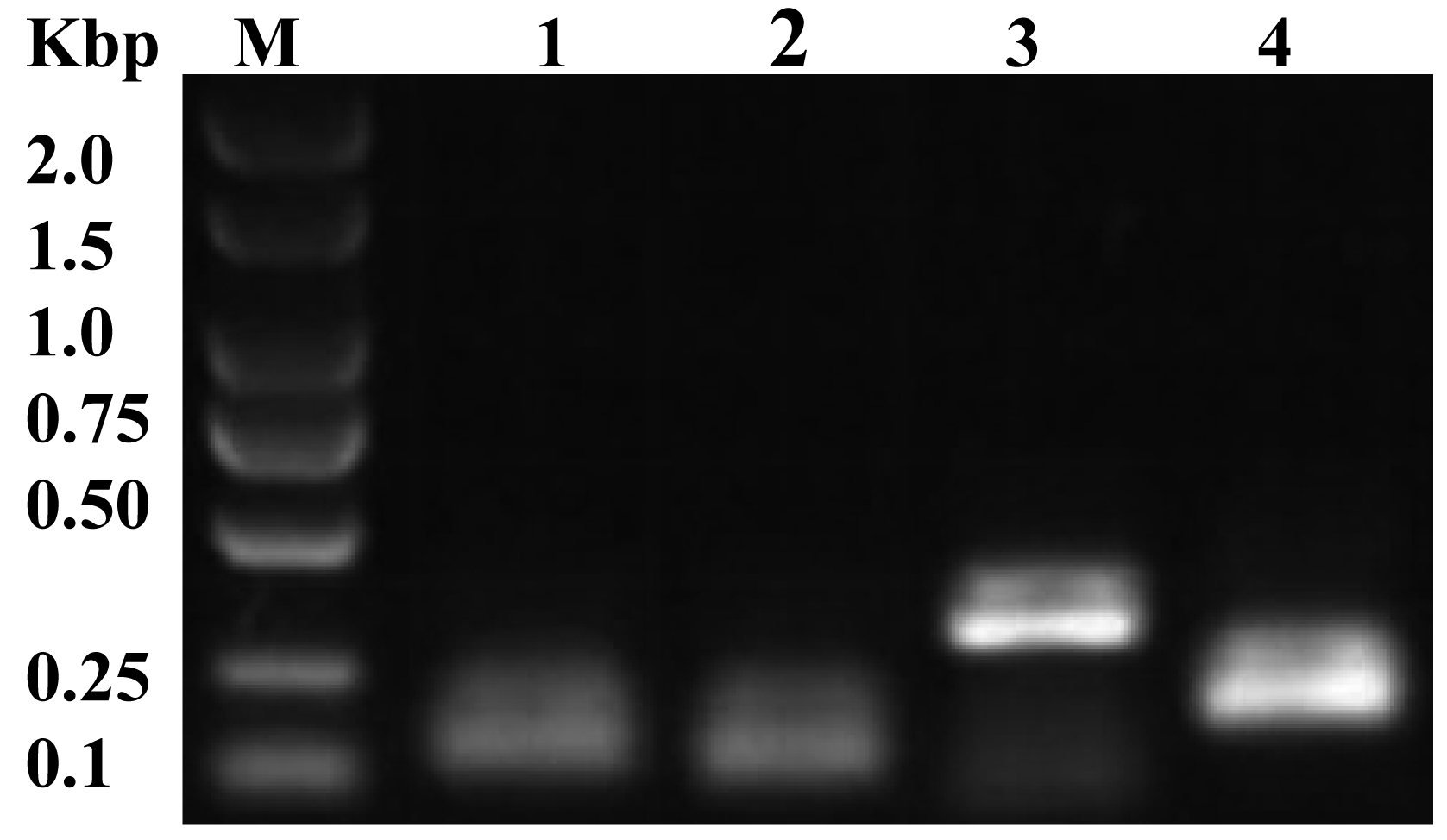


**Supplementary Fig. S2** Identification of the plasmid pNZ8148-PnisA-gfp-P15-nisR-nisK, pNZ8148-PnisA-gfp-P18-nisR-nisK, pNZ8148-PnisA-gfp-P23-nisR-nisK, and pNZ8148-PnisA-gfp-P25-nisR-nisK by PCR assay. Lane1-4: the expected size of the amplified fragment with the specifical primers PG1-P15-F/PG1-P15-R, PG1-P18-F/PG1-P18-R, PG1-P23-F/PG1-P23-R, and PG1-P25-F/PG1-P25-R was about 101 bp, 92 bp, 278 bp, and 151 bp respectively.
