# Supplementary material for: Construction and optimization of a nisin-controlled expression vector using a pre-screened strong promoter in Streptococcus thermophilus
Source: Front Microbiol. 2025 Aug 18;16:1586348. doi: 10.3389/fmicb.2025.1586348 (PMC12401007; doi:10.3389/fmicb.2025.1586348)
Supplement: Supplementary file 2 [file Supplementary_file_2.docx]

**Construction and Optimization of a Nisin-controlled Expression Vector using Pre-screened Strong Promoter in *Streptococcus thermophilus***

**Yanxin Ye^1^, Ruiting Zhao^2^, Leilei Li^1^, Zhi Li^1^, Yanyan Chen^1^*, Zhenshang Xu^2*^**

^1^ School of Life Science and Engineering, Henan University of Urban Construction, Pingdingshan, 467036, Henan, P.R. China;

^2^ School of Bioengineering, Qilu University of Technology, Shandong Academy of Science, Jinan, 250353, Shandong, P.R. China

**^∗^Corresponding author**: Yanyan Chen, E–mail: [20201006@huuc.edu.cn](mailto:yeyanxin2018@huuc.edu.cn)

Zhenshang Xu, E–mail: [xuzhenshang@126.com](mailto:xuzhenshang@126.com)

**Table legends:**

**Table S1:** Different promoter gene sequences pre-screened by our laboratory.

**Table S2:** The specific primers used for this work.

**Table S3:** Expression levels of GFP in different recombinant *S. thermophilus* ATCC19258*, L. plantarum* WCFS1, and *E. faecium* ATCC19434 after induction with 25 ng/mL nisin for 3 h, respectively.

**Table S4:** Expression levels of GFP in different recombinant strains containing the recombinant vector by replacing different promoters after induction with 2500 ng/mL nisin for 3 h when the cells concentration reached 0.5 at OD_600nm_.

# Supplementary Table S1

Table S1 Different promoter gene sequences pre-screened by our laboratory

| **Promoter** | | **Sequence（5’-3’）** |
| --- | --- | --- |
| 15 | GATTGTCGTACCCCTAGGTTTAACCTAGGGGTATTTTTAAATTGAATTTTCAGAAATTAGTGATAAAATAGATAGTAATAAGTATTAAA*AGGAGG*AATCTT |  |
| 18 | AAAAACCGATAAAACTTTTCAAATTTAAAGCCATTTATAATTCTTTGTGTTATAATGAAAATGTTAAATATATAAA*AGGAGT*ATCAGGAATA |  |
| 23 | ACCGACTGTCGGTTGTTAATTGATGTGAAACATCAAAAGAATATTGCACGTAAAACTAAAAAGAAGCGGACATAGGATTCGATTAAGATGAGCTAAACGTCCAATCTTTGATAGAATAAAATAAACAAAAAAAGAAAGGAACAAGGGCACTCGTATTCACTAAAACTGAATACGGGCTACGGACTTGGTCGAAAAGATAGTTTTTCCTAGAAGCTGACGCTTCTTCGTCTAAACTCCTATTTTGACTGTGTCCGCTTAACGCCCTTAATATCTTAATT |  |
| 25 | GAATGGAGCCTGAGTGATTTTGGTTCTTTTCTTTATTGTTCTTTTCTTTATTGTTCTGTAAAGGTTGTTCTTAAAAAATAGGACTTAAGTTCCACACATAAAGCTTAAAAATATGGTAAAATAAGAAGTAAACTAACACTTT*GGAGAG*AAA |  |

# Supplementary Table S2

Table S2 The primers used for this study

| **Primers** | **Sequences (5’-3’)** |
| --- | --- |
| GFP-NcoⅠ-F | TAAATTATAAGGAGGCACTCACCATGAGCAAAGGAGAAGAACTTTT |
| GFP-HindⅢ-R | TTCTAATTTTGGTTCAAAGAAAGCTTCTAGTATAGCTCATCCATGC |
| PN-gfp-F | ACCAAGGCTTGAAACGTTC |
| PN-gfp-R | TTTCTAATTTTGGTTCAAAGA |
| (pNZ8148-gfp)-P_nisR_-F | AAGGAGGCACTCACCATGGCCGGCTTTAGGTATAGTGTG |
| (pNZ8148-gfp)-P_nisR_-F | AGTTCTTCTCCTTTGCTCATCACTTTACCTCCGATTGTTTAG |
| PG1-F | GTGTATAAAATTTTAATAG |
| PG1-R | ACCAAGGCTTGAAACGTTC |
| PG1-P15-F | GAACGTTTCAAGCCTTGGTGATTGTCGTACCCCTAGGTT |
| PG1-P15-R | CTATTAAAATTTTATACACAAGATTCCTCCTTTTAATAC |
| PG1-P18-F | GAACGTTTCAAGCCTTGGTAAAAACCGATAAAACTTTTC |
| PG1-P18-R | CTATTAAAATTTTATACACTATTCCTGATACTCCTTTTA |
| PG1-P23-F | GAACGTTTCAAGCCTTGGTACCGACTGTCGGTTGTTAAT |
| PG1-P23-R | CTATTAAAATTTTATACACAATTAAGATATTAAGGGCGT |
| PG1-P25-F | GAACGTTTCAAGCCTTGGTGAATGGAGCCTGAGTGATTT |
| PG1-P25-R | CTATTAAAATTTTATACACTTTCTCTCCAAAGTGTTAGT |

# Supplementary Table S3

Table S3 Expression levels of GFP in different recombinant *S. thermophilus* ATCC19258*, L. plantarum* WCFS1, and *E. faecium* ATCC19434 after induction with 25 ng/mL nisin for 3 h, respectively.

| **Recombinant Strains** | **Different treatment groups** | **Mean ± SD (RFU)** | **t-value** | **df** | **p-value** | **Significance** |
| --- | --- | --- | --- | --- | --- | --- |
| 1. ***thermophilus***   ATCC19258 | No nisin induction | 15160.72 ± 707.23 | -28.15 | 2.00 | **<0.0001** | *** |
|  | induced with 25 ng/mL nisin | 122170.23 ± 14142.87 |  |  |  |  |
| 1. ***Plantarum***   WCFS1 | No nisin induction | 31604.21 ± 2122.92 | -11.03 | 2.00 | **0.0035** | ** |
|  | induced with 25 ng/mL nisin | 83797.62 ± 1942.08 |  |  |  |  |
| ***E. Faecium***  ATCC19434 | No nisin induction | 23607.72 ± 2122.92 | -21.74 | 2.00 | **<0.0001** | *** |
|  | induced with 25 ng/mL nisin | 206759.68 ± 7071.07 |  |  |  |  |

Data analysis was performed using two-tailed Student's test. **Significance markers**: **p < 0.01, *** *P* < 0.001. SD = Standard deviation; df = Degrees of freedom.

# Supplementary Table S4

Table S4 Expression levels of GFP in different recombinant strains containing the recombinant vector by replacing different promoters after induction with 2500 ng/mL nisin for 3 h when the cells concentration reached 0.5 at OD_600nm_.

| **Recombinant Strains** | **Mean ± SD (RFU)** | **t-value** | **df** | **p-value** | **Significance** |
| --- | --- | --- | --- | --- | --- |
| *S. thermophilus* NICE*-*PnisA | 239761.44 ± 10490.81 | - | - | - | - |
| *S. thermophilus* NICE*-*P15 | 385323.37 ± 3107.84 | 32.34 | 3.99 | 0.0003 | *** |
| *S.thermophilus* NICE*-*P18 | 285242.37 ± 3218.36 | 6.47 | 4.00 | 0.0036 | ** |
| *S.thermophilus* NICE-P23 | 207942.31 ± 24425.03 | -1.20 | 3.04 | 0.3095 | ns |
| *S. thermophilus* NICE-P25 | 393186.29 ± 9084.74 | 29.03 | 3.99 | 0.0004 | *** |

Data analysis was performed using two-tailed Student's test. **Significance markers**: **p < 0.01, *** *P* < 0.001, and ns indicates non-significant statistical differences. SD = Standard deviation; df = Degrees of freedom.
